# Effects of Compound Probiotics on Cecal Microbiota and Metabolome of Swine

**DOI:** 10.3390/ani13061006

**Published:** 2023-03-10

**Authors:** Jie Li, Hongyue Li, Yi Zhou, Hongwei Xiang, Muze Lv, Bo Ruan, Zongyi Bo, Haixiao Shen, Fazhi Xu, Yafeng Huang, Liang Li, Pei Sun

**Affiliations:** 1 College of Animal Science and Technology, Anhui Agricultural University, Hefei 230036, China; 2 Joint International Research Laboratory of Agriculture and Agri-Product Safety, The Ministry of Education of China, Yangzhou University, Yangzhou 225009, China; 3 College of Veterinary Medicine, Nanjing Agricultural University, Nanjing 210095, China

**Keywords:** compound probiotics, swine, intestinal microbiota, metabolomics

## Abstract

**Simple Summary:**

In this study, we used a new mixture to generate probiotics and significantly reduced the relative abundance of Paraprevotella and Phascolarctobacterium, and the group fed with this mixture had higher relative abundance of Prevotella, Faecaliberium, and Gemmiger. These had a big impact on the metabolic pathways. The aim of our study was to use 16S rRNA gene sequencing and untargeted metabolomics to assess how the additive of special probiotics’ mixture influenced the swine microbiome and to confirm whether such a proceeding can reduce the risk of some health disturbances’ appearance.

**Abstract:**

Complex probiotics are made from various single probiotics mixed in scientific formula. The long-term intake of different probiotics is beneficial to maintain the intestinal microecological balance, inhibiting harmful pathogenic flora and facilitating organism health. Based on the limited research on intestinal flora and related metabolites after the long-term intake of the probiotic complex, in this study, 16S rRNA gene sequencing and untargeted metabolomics were used to further investigate the effects of the probiotic complex on the intestinal flora and metabolome of pigs. The results demonstrated that the content of flora in the intestinal tract or metabolites of pigs varied greatly and was related to cellular metabolic pathways after the long-term feeding of complex probiotics. This study provides a valuable theoretical basis for farmers to raise pigs scientifically and healthily.

## 1. Introduction

Probiotics that contain one or more live-specific microorganisms can provide several beneficial effects in the host body. They can inhibit the activity of pathogenic microflora invading the intestines and reduce harmful pathogenic bacteria. Probiotics have been used to overcome the problems of harmful gas emissions from swine, post-weaning diarrhea in swine, and the increase in antibiotic-resistant pathogens [1,2,3]. The main probiotics used in animal production are lactic acid bacteria, yeast, and bacillus. Among them, *Bacillus subtilis* (*B. subtilis*) can enhance and add beneficial intestinal flora, inhibit harmful intestinal bacteria, improve swine meat quality, and reduce skatole [4]. *Bacillus coagulans* (*B. coagulans*) can promote the decomposition of protein and various carbohydrates, and reduce flatulence and abdominal distension [5]. Yeast can alleviate the symptoms of post-weaning infection with ETEC and reduce the incidence of diarrhea [6]. Because antibiotics can no longer be used as feed additives in many countries [7], probiotics have attracted much attention as some of the most effective alternatives to antibiotics. However, the research reports on the action mechanism of probiotics on the gastrointestinal tract of swine are relatively few. For example, some scientists demonstrated that the selected *Lactobacillus rhamnosus* strain promoted the activation of the phosphorylated epidermal growth factor receptor (EGFR) independent Akt in the IPEC-J2 cell model infected by the enterotoxin-producing *Escherichia coli* K88 [8]. It was found that the dietary supplementation of *Clostridium butyricum* can improve the intestinal barrier function of weaned piglets attacked by the enterotoxin-producing Escherichia coli K88. *Clostridium butyricum* enhanced the intestinal barrier function of weaned piglets, and inhibited the signal pathway of the apoptosis-related punctate protein-dependent NLRP88 inflammatory corpuscles in weaned piglets after the ETEC K3 stimulation [9]. Moreover, some papers confirmed that intestinal epithelial cells are the targets of the infectious gastroenteritis (TGE) virus (TGEV) infection. Bacillus subtilis is a probiotic with excellent antimicrobial properties. *Bacillus subtilis* and a surfactant can prevent the infectious gastroenteritis virus from entering intestinal epithelial cells in vitro [10]. In addition, the 16S rRNA gene sequencing was used for microbial analysis, and it was verified that one of the mechanisms by which Saccharomyces cerevisiae can improve the performance of pigs was to change the composition of the intestinal microflora; this reaction could be enhanced by supplementing probiotics in the early postpartum period [11]. The regulation of intestinal flora and intestinal metabolites in swine needs to be explored.

The intestinal microflora is an important part of the diversified intestinal ecosystem. Factors such as changes in the host diet, stimuli, and drugs may change the physiological activities of the host by changing the microbial composition [12]. Studies on metabolites associated with the intestinal microbiota contribute to a better understanding of the effects of lifestyle and dietary factors on the disease [13].

In recent years, fecal microbiology–metabolomics has attracted more and more attention, and has obtained many valuable results in the intestinal microbial metabolism [14]. Feces are the final product of the digestive process in the intestinal tract. Usually, the experimental research object is the feces excluded from the body. However, intestinal digestion is a long process; it may not fully reflect the effects of probiotics on the gastrointestinal tract. There are few research reports on collecting the contents of different parts of the pig intestine, such as the duodenum and cecum for a microbiological analysis.

In China, the pig breeding industry is relatively developed, and there is a high demand for live pigs on the market. The health of piglets is also a world concern. Considering the public health problem, China prohibits the use of antibiotics as feed additives in feed. Therefore, probiotics have attracted more and more attention in the livestock industry. Here, in this study, a complex of probiotics was used as feed additives to feed swine, and high-throughput sequencing and nontargeted metabolomics were used to analyze the differences of fecal flora and metabolites in the duodenum, cecum, and anus, in order to investigate the effects of probiotics on the intestinal microbes and metabolism of swine.

## 2. Materials and Methods

### 2.1. Experimental Materials

The compound probiotics (*B. subtilis* ≥ 1.0 × 10^9^ CFU/g; *B. coagulans* ≥ 1.0 × 10^8^ CFU/g; yeast ≥ 5.0 × 10^7^ CFU/g) used in this study were provided by China Fujian Luodong Biotechnology Co, Ltd., Zhangpu City, China, Based on the physiological and nutritional needs of the swine, the diet was formulated as shown in Supplementary Material (Table S1).

### 2.2. Animals and Experimental Design

The experiment was started on a farm in Fuyang, Anhui. All the animal experiments were accepted by the Animal Care and Ethics Committee of the Anhui Agricultural University (permit number: SYXK 2016-007) and followed the Guiding Principles for Biomedical Research Including Animals. Forty 45-day-old swine ([Large White ♂ × Landrace ♀] × Large White ♂, 15.5 ± 0.5 kg BW) were used. Two experimental diets were devised: the control diet (CON) and the control diet supplemented with 100 mg/kg compound probiotics (MixP). Each treatment contains four replications and five swine at 45 days of age for each replication. All swine were given access to water and fed ad libitum. Swine were housed separately in individual pens. The experiment lasted for 30 days. After the trial ended, 8 swine (without feeding for 12 h, with one piglet per pen and four piglets in each group) were randomly selected and euthanized with sodium pentobarbital (50 mg/kg BW). Fecal samples were collected from the duodenum, cecum, and anus. Samples were frozen immediately in liquid nitrogen, stored at −80 °C, and then transported by dry ice to Huada Company (Huada, Shenzhen, China) for measurement.

### 2.3. DNA Extraction and 16S rRNA Gene Amplification Sequencing

Sequencing was finished in the Huada Gene Company, Shenzhen, China. Samples were extracted by the QIAamp DNA Stool Mini Kit (Beijing Genomics Institute, Shenzhen, China) according to the manufacturer’s instruction. The DNA was amplified by using the 515F-805R primer set (515F: 5′-CCTACGGGNGGCWGCAG-3′ and 805R: 5′-GACTACHVGGGTATCTAATCC-3′) [11], which targeted the V4 region of the bacterial 16S rDNA. The PCR amplification used the Phusion High-Fidelity Master mix (Beijing Genomics Institute, Shenzhen, China), and the condition was 98 °C for 1 min. It was denatured at 98 °C for 10 s, annealed at 50 °C for 30 s, extended at 72 °C for 30 s, 30 cycles, and finally extended at 72 °C for 5 min. The samples were purified using Agencurt AMpure XP beads, dissolved in Elution buffer, and labeled. The Agilent 2100 Bioanalyzer system was accustomed to testing the fragment range and concentration of the library. The library that passed the test was sequenced using the Illumina Hise-q2500 platform, and the sequencing type was PE250.

### 2.4. Sequence Analysis

The data were filtered using the readfq software independently developed by Huada, and the QIIme software was used for quality control. The software FLASH (Fast Length Adjustment of Short Reads, v1.2.11) was used to attain the Tags of the high-temperature range. USEARCH (v7.0.1090) was used for UPARSE clustering under the 97 similarity. The chimeras generated by the PCR amplification were removed from the OTU representative sequence using UCHIME (v4.2.40). The OTU representative sequences were compared to the Greengene database for species annotation by RDP Classic FER (v2.2) with a confidence threshold of 0.8. The alpha and beta diversity were calculated at the OTU level using MOTHUR (v1.31.2) and QIIME (v1.8.0), multiple times. The Wilcoxon Rank Sum Test was used to compare the Alpha diversity of intestinal flora in different groups, and the unweighted UniFrac (unweighted UniFrac)-based principal component analysis (PCoA) was used to measure the beta diversity among groups.

### 2.5. Metabolite Extraction

The extraction method of metabolites mainly refers to the previous research reports. Briefly, 25 mg tissues were weighed and extracted by directly adding 800 µL of the precooled extraction reagent (methanol: acetonitrile: water (2:2:1, *v*/*v*/*v*)); the internal standard mix was added for the quality control of the sample preparation. After homogenizing for 5 min using TissueLyser (Beijing Genomics Institute, Shenzhen, China), samples were then sonicated for 10 min and incubated at −20 °C for one hour. Samples were centrifuged for 15 min at 25,000 rpm at 4 °C, and the supernatant was then transferred for vacuum freeze drying. The metabolites were resuspended in 600 µL of 10% methanol and sonicated for 10 min at 4 °C, after centrifuging for 15 min at 25,000 rpm, and the supernatants were transferred to autosampler vials for the LC-MS analysis. A quality control (QC) sample was prepared by pooling the same volume of each sample to evaluate the reproducibility of the whole LC-MS analysis.

### 2.6. LC-MS/MS Analysis

This experiment used a Waters 2D UPLC (Beijing Genomics Institute, Shenzhen, China) tandem Q Exactive high resolution mass spectrometer (Beijing Genomics Institute, Shenzhen, China) for the separation and detection of metabolites. The mass spectrometric settings for positive/negative ionization modes were as follows: spray voltage—3.8/−3.2 kV; sheath gas flow rate—40 arbitrary units (arb); aux gas flow rate—10 arb; aux gas heater temperature—350 °C; capillary temperature—320 °C. The full scan range was 70–1050 *m*/*z* with a resolution of 70,000, and the automatic gain control (AGC) target for MS acquisitions was set to 6 × 10³ with a maximum ion injection time of 100 ms. The top 3 precursors were selected for subsequent MSMS fragmentation with a maximum ion injection time of 50 ms and resolution of 17,500; the AGC was 1 × 10^5^. The stepped normalized collision energy was set to 20, 40, and 60 eV.

### 2.7. Metabolomics’ Analysis

The LC-MS/MS data processing was performed using The Compound Discoverer 3.1 (Beijing Genomics Institute, Shenzhen, China) software, primarily containing the peak extraction, peak alignment, and compound identification. Data pre-processing, statistical analysis, metabolite classification notes, and functional notes were performed using the self-developed metabolomics R package metaX [12] and the metabolome bioinformatic analysis pipeline. The analyses of metabolites were verified by the BGI self-built standard library, and the mzCloud and ChemSpider (HMDB, KEGG, LipidMaps) databases.

### 2.8. Statistical Analysis

Data pre-processing, statistical analysis, metabolite classification annotations, and functional annotations were performed using the self-developed metabolomics R package metaX; the *p*-value is obtained through the Wilcoxon Rank Sum Test.

## 3. Results

### 3.1. Effect of Feeding Probiotics on Intestinal Microbiota

**Figure 1:**
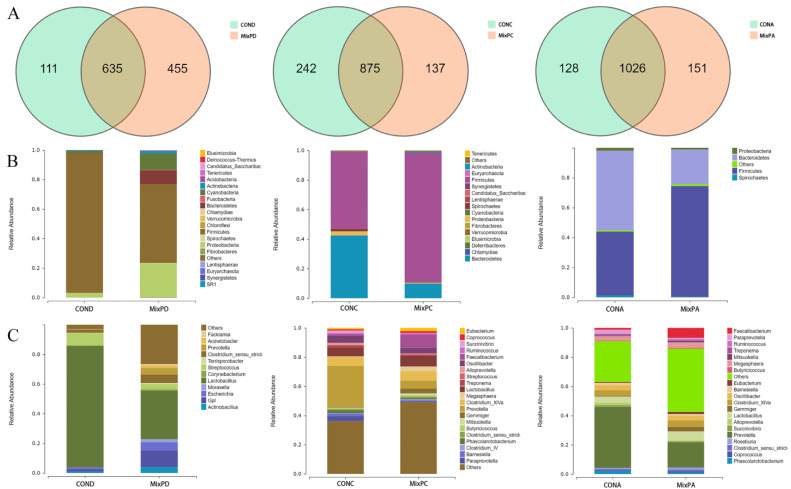
Summary of compound probiotics regulating intestinal microbial community in swine. (**A**) Venn chart of duodenum (**left**), cecum (**middle**), and feces (**right**). (**B**) Percentage composition of the top 10 predominant phyla in the duodenal (**left**), cecal (**middle**), and fecal content (**right**). (**C**) Percentage composition of the top 10 predominant genus in the duodenal (**left**), cecal (**middle**), and fecal content (**right**).

Using 16S rRNA sequencing, the effect of complex probiotics on cecal microflora is shown in Figure 1. A total of 1201, 1254, and 1305 operational taxonomic units (OTUs), respectively, were identified in the duodenal, cecal, and fecal contents of the swine. A total of 455, 137, and 151 unique OTUs were observed in the duodenum, cecum, and feces, respectively (Figure 1A). At the level of the phylum and genus, all content samples from the experimental groups and control groups showed almost the same community structure. The abundance of each species in the duodenal, cecal, and fecal contents was varied (Figure 1B,C). This indicated that the probiotics interfered with the species and quantity of the intestinal flora in swine.

**Figure 2:**
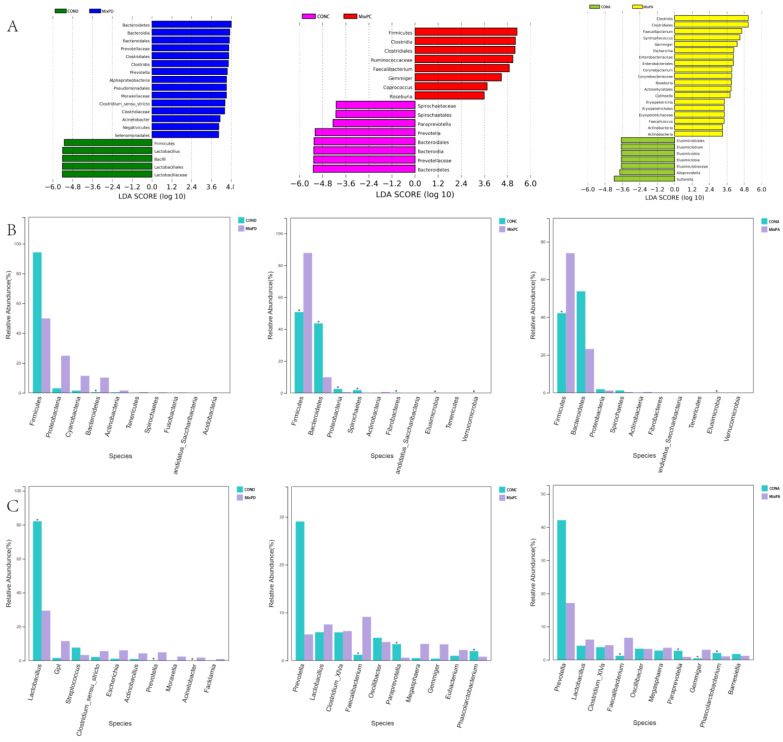
LEfSe analysis of the relative abundance of predominant microflora in swine. (**A**) LEfSe analysis of duodenum (**left**), cecum (**middle**), and feces (**right**). (**B**) Comparison of key species differences of duodenum (**left**), cecum (**middle**), and feces (**right**) at the phylum level. (**C**) Comparison of key species differences of duodenum (**left**), cecum (**middle**), and feces (**right**) at the genera level.

From the effect size measurements’ (LEfSe) analysis (Figure 2A), Bacteroidetes, Prevotellaceae, Clostridiales, Alphaproteobacteria, Moraxellaceae, etc., were the dominant genera in the duodenal contents; Clostridiales, Ruminococcaceae, Faecalibacterium, Gemmiger, Coprococcus, Roseburia dominated the cecal contents; Clostridiales, Faecalibacterium, Gemmiger, Syntrophococcus, etc., dominated the fecal contents. The species with the most abundance among the top 10 were selected to test the significance of the difference (Figure 2B,C). The compound probiotics dramatically decreased the relative abundance of Bacteroidetes, Spirochaetes, Fibrobactere, lusimicrobia, Verrucomicrobia, Paraprevotella, and Phascolarctobacterium in the cecum; Elusimicrobia, Paraprevotella, and Phascolarctobacterium in the feces. The swine of the experimental group had a higher relative abundance of Bacteroidetes, Prevotella, and Acinetobacter in the duodenum; Firmicutes and Faecalibacterium in the cecum; and Firmicutes, Faecalibacterium, and Gemmiger in the feces than the swine of the control group.

### 3.2. Effect of Feeding Probiotics on Intestinal Metabolites

**Figure 3:**
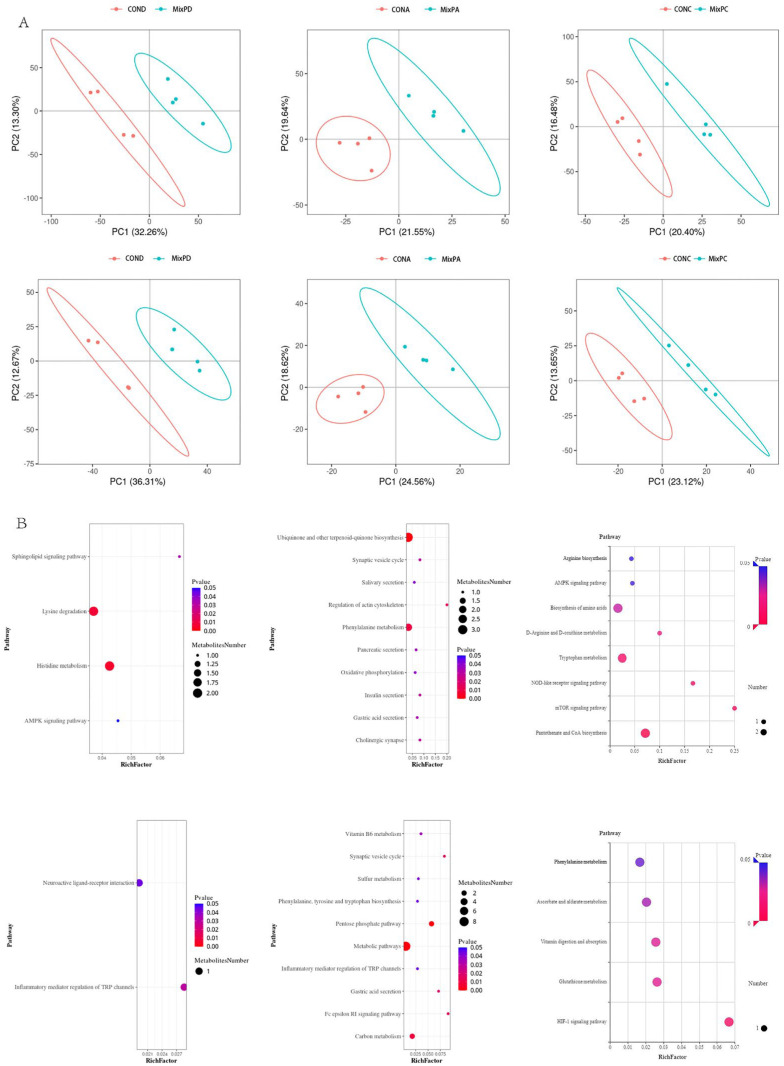
Metabolic analyses on the duodenal (**left**), cecal (**middle**), and fecal content (**right**). (**A**) Cluster analysis based on the metabolites in positive (**up**) and negative (**down**) ion modes using the partial least-squares discrimination method. (**B**) Cecal metabolomics’ pathway analysis of swine that received the compound probiotics diet (MixP) in comparison with the control diet (Ctrl). The colors and sizes of the shapes represent the effects of the compound probiotics treatments on sample metabolism relative to the control treatments; larger red shapes indicate a greater effect on the pathway.

We used a partial least squares discriminant analysis model to test the relationship between metabolite levels and sample types. Samples from the experimental and control groups in duodenum, cecum, and feces were grouped into markedly different clusters in both ion modes, indicating significant changes in the gastrointestinal metabolic profile after the administration of the complex probiotic (Figure 3A). To reveal the potential function of the differential metabolites, we performed a pathway enrichment analysis. Differential metabolites in duodenum, cecum, and feces observed that the differential metabolites were significantly enriched in pathways such as lysine degradation, histidine metabolism, phenylalanine metabolism, the pentose phosphate pathway, the HIF-1 signaling pathway, and the mTOR signaling pathway (Figure 3B,C).

### 3.3. Correlation between the Cecal Microbiota and Metabolome

**Figure 4:**
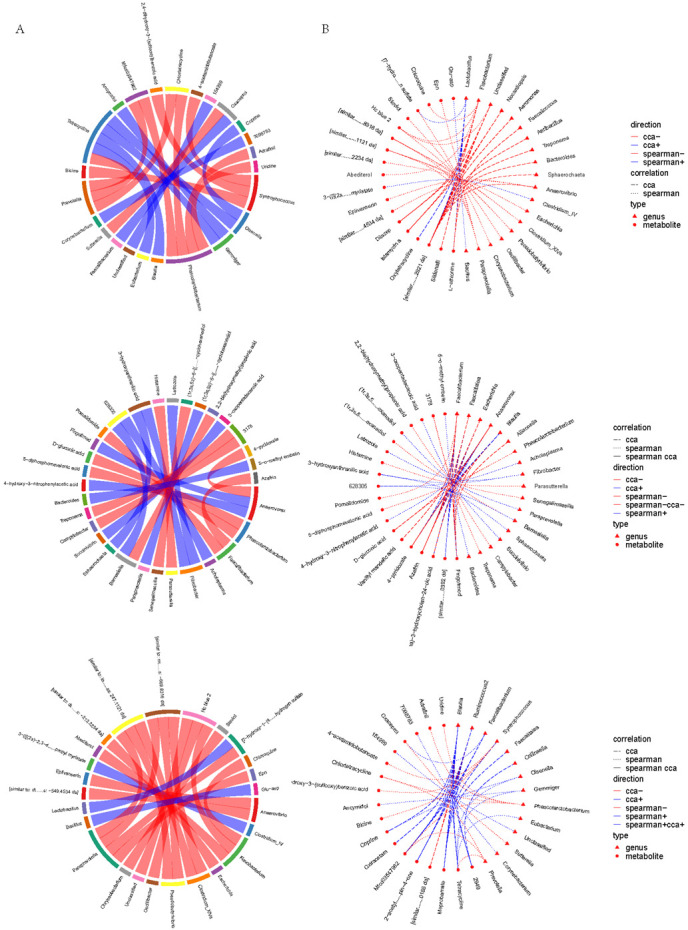
Correlation between microbial species and metabolome detection results of the duodenal (**up**), cecal (**middle**), and fecal content (**down**). (**A**) Chord diagram of correlation between the co-expression clusters of metabolites and microbial groups. (**B**) Network diagram of correlation between the co-expression clusters of metabolites and microbial groups.

The rank correlation coefficient between each differential metabolite and each microbial group was calculated to analyze the correlation between them (Figure 4). The analysis revealed a high correlation between microorganisms and certain metabolites in the duodenum, cecum, and feces (*p* < 0.05). For example, the relative abundances of the genera Gemmiger were negatively correlated with Ancymidol (*p* < 0.05). The relative abundances of the genera Phascolarctobacterium were negatively correlated with *Dgluconic acid* (*p* < 0.05). These data indicate that the specificity of the intestinal microbial composition affects the intestinal metabolites to some extent.

## 4. Discussion

Antibiotics as growth promoters (AGPs) in animal feed have been banned from animal production in EU countries. For example, since September 1999, the European Union (EU) has banned the use of anti-microbial growth promoters (AGPs), carbadox and quinol [15]. Probiotics, as a substitute for antibiotics, are widely used in the production of animal feed, and have achieved some significant results in recent research. Some probiotics can be used in different stages of pig production and have been proved to be effective in preventing, controlling, and treating infection [16]. The research demonstrates that probiotics’ combination can improve body weight, digestibility, and feed efficiency [17]. In this experiment, the contents of the duodenum, cecum, and feces of pigs fed with probiotics were extracted and studied. The experimental results demonstrated that the intestinal digestive function of pigs fed with probiotics was improved; in particular, the content of some intestinal microflora was significantly different, which is beneficial to understanding the influence of probiotics on the intestinal digestion of pigs.

There are a large number of microbial populations in the cecum of single-stomach animals, which have a huge impact on the metabolism of host nutrients and intestinal health. In the ‘hindgut nutrition’ of single-stomach animals, the role of cecum microorganisms cannot be ignored. The microbial composition in the pig cecum significantly affects the host’s health, immunity, nutritional digestion, and feeding requirements [18]. This study demonstrated that the effect of the probiotic mixture on the composition of the mucin carbohydrate secreted by Brunner’s gland in the duodenum of growing–finishing pigs was studied by routine histochemistry. The probiotics supplement affected the composition of the mucin carbohydrate secreted by Brunner’s gland in growing–finishing pigs. These changes can effectively affect the gastrointestinal function and animals’ health [19]. In this experiment, the same batch of 45-day-old American backcross binary healthy weaned piglets were selected; they had a relatively complete digestive system and were fed with compound probiotics. The species and quantity of bacterial flora in the experimental groups were significantly different from the control groups.

As an important part of the intestine, the important activities of all intestinal microflora are closely related to metabolism, nutrition, immune response, and health [20]. We observed a high diversity in the microbial communities, similarly to other authors. For example, the abundance of Para Prevotella is closely related to the growth performance of pigs. It is found that there is a positive correlation between Para Prevotella and the reduction of the feed/meat ratio [21]. In this experiment, compared with the significant change of this genus, the difference between the probiotics group and control group is the inclusion of Prevotella, Faecalibacterium, Gemmiger, Paraprevotella, etc. Bacillus faecalis has an anti-inflammatory effect on the gastrointestinal tract. It is reported that the Bacillus faecalis can prevent the activation of NF-κB and the production of IL-8 in epithelial cells by secreting bioactive factors [22]. In their research, they found that the addition of the SPL supplement after weaning may reduce the expression level of proinflammatory cytokines by increasing the abundance of Gemmiger [23]. These studies demonstrate that adding compound probiotics can help affect the growth efficiency and body function of the pig population.

Intestinal flora has a big impact on intestinal biochemical reactions [24]. Metabolomics can further explore how intestinal flora affects the host metabolism and changes in daily metabolomics. Studies have demonstrated that the measurement of the fecal metabolic profile has been proved to be repeatable and has provided important results on the impact of intestinal microorganisms on host metabolism [25,26]. LC-MS/MS mass spectrometry was used to study the effects of compound probiotics on the contents of different intestinal segments and the contents of the feces of pigs. In animal feed, Bacillus subtilis is the most commonly used probiotic strain [27,28,29,30,31]. The compound probiotics used in this experiment are composed of lactic acid bacteria, yeast, bacillus subtilis, and bacillus coagulase. The results demonstrated that probiotics had a significant effect on the metabolism of pigs. In this experiment, we found that different metabolites were enriched in lysine degradation, histidine metabolism, phenylalanine metabolism, pentose phosphate pathway, HIF-1 signal pathway, and mTOR signal pathway.

Histidine is a semi-essential amino acid, which is mainly produced by the intestinal microbial metabolism. According to the results of this experiment, it is speculated that the probiotics complex may affect the metabolism of histidine by affecting the production of histidine. Some studies have found that histamine can be produced during histidine metabolism and inhibit the tumor necrosis factor-α (TNF-α) [32]. It also prevents bacterial translocation. Histidine has been disclosed for its role in the treatment of inflammatory reactions, oxidative stress, and metabolic disorders [33]. Phenylalanine is an essential amino acid and must be obtained from the diet. Phenylalanine is usually decomposed into a variety of active substances by related enzymes in the body, including tyrosine, which affects the physiological activities of animals. It plays an important role in regulating the glucose and lipid metabolism of the body [34]. The pentose phosphate pathway connects the carbohydrate and fatty acid metabolism, malnutrition, nucleotide synthesis, and antioxidant defense [35]. HIF-1 is composed of the HIF-1α and HIF-1β combined formation, which can control the survival of immune cells and participate in many inflammatory diseases, such as bacterial and viral infections, immune diseases, and macrophage metabolism [36]. The mTOR signal pathway also plays an important role in controlling the production of many nutrients and energy metabolism. mTORC1 can control the expression of orexin (NPY, AgRP) to affect the food intake by activating PPAR-γ, and also promotes fat production, reduces fat decomposition, and promotes glucose uptake through the mTORC2-Akt activation [37]. A lot of previous research results are mostly focused on the research of a specific signal pathway. In this study, several important signal pathways with significant differences are obtained through the pathway enrichment analysis. Although the results obtained by our discussion are different from others, which may be due to high individuals’ variability, or because we chose the duodenum, cecum, and feces of pigs for the experiment, this provides a theoretical basis for later scholars on which to conduct more comprehensive research. Therefore, this study may further demonstrate that the long-term and healthy feeding of complex probiotics is beneficial for improving the digestive function of pigs’ intestines.

## 5. Conclusions

The results demonstrated that the compound probiotics dramatically decreased the relative abundance of Paraprevotella and Phascolarctobacterium; the group fed with compound probiotics had a higher relative abundance of Prevotella, Faecalibacterium, and Gemmiger. Differential metabolites were enriched on metabolic pathways such as lysine degradation, histidine metabolism, phenylalanine metabolism, the pentose phosphate pathway, the HIF-1 signaling pathway, and the mTOR signaling pathway. The results of this study will provide basic data for scientific research and regulatory management of intestinal flora in swine production.

## Data Availability

This study contains all datasets that were proved in the article.

## Supplementary Materials

The following supporting information can be downloaded at: https://www.mdpi.com/article/10.3390/ani13061006/s1, Table S1: Composition and nutrient levels of basal diets (air-dry basis) %.
