# Supplementary material for: Effects of Compound Probiotics on Cecal Microbiota and Metabolome of Swine
Source: Animals (Basel). 2023 Mar 10;13(6):1006. doi: 10.3390/ani13061006 (PMC10044668; doi:10.3390/ani13061006)
Supplement: Supplementary file 1 [file animals-13-01006-s001.zip › animals-2225176-supplementary.pdf]

Supplementary Table S1 Composition and nutrient levels of basal diets (air-dry basis) %

Items	Content
Corn	57.46
High protein soybean meal	8.29
Fermented soybean meal	5
Imported steam fishmeal	2
Enzymatic protein	2
Mixed wheat bran	5
Wheat middlings	5.63
Soybean expanded	5
Soybean oil	1.46
CaHPO4	1.16
Five Grain Acid	0.3
Micromellitic acid	0.2
Glucose	2.5
Premix1)	4
Total	100
Nutrient levels2)	Content
ME(MJ/kg)	13.8
CP	18.00
Ash	5.01
EE	4.56
C-fiber	2.69
Ca	0.65
Total P	0.54
Salt	0.80
Total lys	1.2646
Met	0.4497
Met+Cys	0.7497
Thr	0.8837
TRP	0.2970

[1] The premix provided the following per kilogram of the diet: VA 16000 IU, VD 34000 IU, VE 100 IU, VK3 0.5 mg, VB1 2 mg, VB2 4.5 mg, VB6 7 mg, VB12 0.03 mg, biotin 0.2 mg, folic acid 10 mg, nicotinic acid 30 mg, pantothenic acid 22 mg, Lys-HCl 5.73 g, Lthreonine 2.35 g, DL-Met. 1.59 g, L-tryptophan 0.58 g, Fe(FeSO4) 85 mg ,Cu(CuSO4) 100 mg, Mn(MnSO4) 0.3 mg, and I(CaI2) 0.14 mg.

[2] Ash, EE, C-fiber, DM, CP, Ca, Salt and TP were measured values, while the others were calculated values.
